# Using Second Measurement of De Ritis Ratio to Improve Mortality Prediction in Adult Trauma Patients in Intensive Care Unit

**DOI:** 10.3390/diagnostics12122930

**Published:** 2022-11-24

**Authors:** Wei-Ti Su, Cheng-Shyuan Rau, Sheng-En Chou, Ching-Hua Tsai, Peng-Chen Chien, Ching-Hua Hsieh

**Affiliations:** 1 Department of Trauma Surgery, Kaohsiung Chang Gung Memorial Hospital, Chang Gung University College of Medicine, Kaohsiung 83301, Taiwan; 2 Department of Neurosurgery, Kaohsiung Chang Gung Memorial Hospital, Chang Gung University College of Medicine, Kaohsiung 83301, Taiwan

**Keywords:** trauma, intensive care unit (ICU), mortality, aspartate aminotransferase (AST), alanine aminotransferase (ALT), De Ritis ratio (DRR)

## Abstract

The De Ritis ratio (DRR), the ratio of serum levels of aspartate aminotransferase/alanine aminotransferase, has been reported to be a valuable biomarker in risk stratification for many liver and non-liver diseases. This study aimed to explore whether the inclusion of DRR at the date of intensive care unit (ICU) admission or days after ICU admission improves the predictive performance of various prognosis prediction models. This study reviewed 888 adult trauma patients (74 deaths and 814 survivors) in the trauma registered database between 1 January 2009, and 31 December 2020. Medical information with AST and ALT levels and derived DRR at the date of ICU admission (1st DRR) and 3–7 day after ICU admission (2nd DRR) was retrieved. Logistic regression was used to build new probability models for mortality prediction using additional DRR variables in various mortality prediction models. There was no significant difference in the 1st DRR between the death and survival patients; however, there was a significantly higher 2nd DRR in the death patients than the survival patients. This study showed that the inclusion of the additional DRR variable, measured 3–7 days after ICU admission, significantly increased the prediction performance in all studied prognosis prediction models.

## 1. Introduction

The serum ratio of aspartate aminotransferase (AST)/alanine aminotransferase (ALT), the so-called De Ritis ratio (DRR)”, has been demonstrated to be a valuable tool in risk stratification for different kinds of liver diseases [1,2,3]. ALT is found predominantly in the cytosol of hepatocytes, while AST is found in the cytosol and mitochondria of hepatocytes as well as in the cells in the brain, kidney, heart, and skeletal muscle [3]; Therefore, the increase in the serum level of ALT indicates parenchymal liver illness with liver-specific dysfunction, while the increase in the serum level of AST suggests systemic involvement other than liver dysfunction. Ischemia-reperfusion injury, oxidative stress, and metabolic disorders can increase serum levels of AST [4,5,6]. Therefore, DRR has also been proposed to be valuable in the diagnosis and risk stratification of many illnesses other than liver diseases, including cancers other than hepatoma [1,7,8,9,10,11,12,13], acute kidney injury [14,15,16], heart diseases [17,18,19], sepsis [20], and coronavirus disease 2019 (COVID-19) [21,22,23,24].

Prognosis prediction models are broadly used in intensive care units (ICU) for risk stratification, quality control, and scientific research [25,26,27,28]. The Trauma Score and Injury Severity Score (TRISS) [29] is generally recommended for trauma patients; however, for patients with critical illness in the ICU, some other prognosis prediction models have been proposed and reviewed in the literature [30,31,32]. Most of these models, such as the Acute Physiology and Chronic Health Evaluation (APACHE) [33], Simplified Acute Physiology Score (SAPS) [34], and Mortality Prediction Model (MPM) [35], are based on data collected on the first day of admission to the ICU. Other models collect data every day throughout the stay in the ICU or for the first 3 days in the ICU, including MPM II at 24 h (MPM_24_ II), MPM II at 48 h (MPM_48_ II), MPM II at 72 h (MPM_72_ II) [36,37], Logistic Organ Dysfunction System (LODS) [38], Multiple Organ Dysfunction Score (MODS) [39], 24 h ICU point system [40], Sequential Organ Failure Assessment (SOFA) [41], and Three-Day Recalibrating ICU Outcomes (TRIOS) [29].

To improve the accuracy of outcome prediction for trauma patients in the ICU, this study was designed to explore whether the inclusion of DRR at the date of admission or days after ICU admission as a variable in these various prognosis prediction models could improve predictive performance. In this study, we found that the inclusion of the 2nd DRR, a value of AST/ALT measured between days 3 and 7 after ICU admission, as an additional variable in all prognosis prediction models, can build models with better predictive performance for mortality.

## 2. Materials and Methods

### 2.1. Study Population and Data Collection

**Figure 1:**
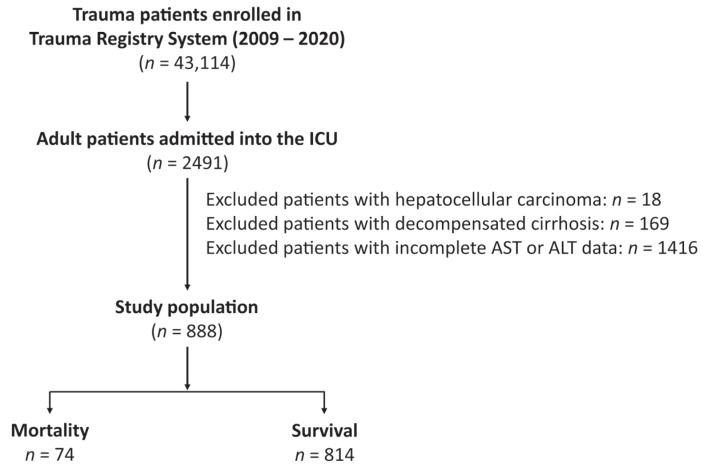
Flowchart demonstrating the inclusion of hospitalized adult trauma patients admitted into the intensive care unit. After the exclusion of patients who had hepatocellular carcinoma, pre-existed decompensated cirrhosis, and lacked AST or ALT data, the study population was grouped into the death (*n* = 74) and survival groups (*n* = 814).

Of 43,114 hospitalized trauma patients by all trauma causes enrolled in the trauma registered database of the Chang Gung Memorial hospital [42,43,44] between 1 January 2009, and 31 December 2020 (Figure 1), 2491 patients aged ≥20 years admitted to the ICU were included. After excluding patients with hepatocellular carcinoma (*n* = 18), pre-existing decompensated cirrhosis (*n* = 169), and those who lacked AST or ALT data (*n* = 1416), we finally included 888 adult trauma patients with critical illness in the study population. Decompensated cirrhosis was defined as the presence of at least one pre-existing complication, including jaundice, ascites, variceal bleeding, or hepatic encephalopathy [45]. Patients’ medical information, which was recorded upon arrival at the emergency department, was retrieved from the registered trauma database, including age, sex, body mass index (BMI), pre-existing comorbidities, vital signs, Glasgow coma scale (GCS) score, abbreviated injury scale (AIS) in different body regions, injury severity score (ISS), and in-hospital mortality. Blood-drawn laboratory data at admission to the ICU included glucose, bicarbonate (HCO_3_), sodium (Na), potassium (K), red blood cell count (RBC), white blood cell count (WBC), neutrophil (%), hemoglobin (Hb), hematocrit (Hct), platelets, international normalized ratio (INR), blood urine nitrogen (BUN), creatinine (Cr), albumin, bilirubin, aspartate aminotransferase (AST), and alanine aminotransferase (ALT). The pre-existing comorbidities recorded included hypertension (HTN), coronary artery disease (CAD), end-stage renal disease (ESRD), cerebrovascular accident (CVA), and diabetes mellitus (DM). The levels of AST and ALT detected upon admission to the ICU were defined as the first measurement of the liver enzymes (1st AST and 1st ALT, respectively), while the levels of AST and ALT detected between days 3 and 7 were defined as the second measurement of the liver enzymes (2nd AST and 2nd ALT, respectively). Therefore, the AST/ALT ratio of the first measurement produced 1st DRR, whereas the AST/ALT ratio of the second measurement indicated the 2nd DRR.

### 2.2. Statistical Analyses

In this study, we used the commercial software SPSS Windows (version 23.0; IBM Inc., Chicago, IL, USA) for all statistical analyses. Categorical data were compared using two-sided Fisher’s exact test or Pearson’s χ^2^ test. Normalization of the distributed continuous data was analyzed using the Kolmogorov–Smirnov test. Non-normally distributed continuous data were compared using the Mann–Whitney U test, with the data expressed as median with interquartile range (IQR) between Q1 and Q3. The predictive performance of 1st and 2nd DDR on patient mortality was determined based on the area under the receiver operating characteristic curve (AUCROC). The score, as well as the variables for different mortality prediction models, including TRISS, MPM II, MPM_24_ II, MPM_48_ II, MPM_72_ II, APACHE II, SAPS II, LODS, MODS, 24 h ICU point system, SOFA, and TRIOS, were retrieved from the trauma-registered database. Logistic regression was used to reconstruct new probability models for mortality prediction with the addition of the 2nd DRR to the variables of different mortality prediction models. A two-tailed *p*-value < 0.05 was considered statistically significant.

## 3. Results

### 3.1. The Patient and Injury Characteristics of the Death and Survival Patients

This study included 74 deaths and 814 surviving patients. There were no significant differences in sex between the death and survival groups (Table 1). Significantly higher rates of pre-existing comorbidities of HTN, CAD, and ESRD were found in patients who died than in those who survived. The death and survival patients presented significant differences in AIS of injuries in the head and abdominal regions but not in other body regions. As shown in Table 2, the patients who died were significantly older than those who survived (*p* < 0.001). The death patients had significantly lower GCS (median [IQR, Q1–Q3], 6 [3–11] vs. 11 [8–15], *p* < 0.001) but higher ISS (25 [19–29] vs. 20 [16–25], *p* < 0.001) than the survival patients. The death patients had significantly higher levels of glucose, BUN, and Cr, but lower HCO3, INR, and albumin levels than the survival patients. Notably, there was no significant difference in the bilirubin levels of the death and survival patients (0.8 [0.5–1.4] vs. 0.8 [0.6–1.1], *p* = 0.826).

Regarding liver enzymes, the day to check the 2nd measurement was at median 5 day (IQR: 4.3–5.9 day). there were no significant differences in the levels of 1st AST, 1st ALT, and 2nd ALT between the death and survival groups. However, there was a significantly higher level of 2nd AST in patients in the death group than those in the survival group (57 [32–107] vs. 39 [26–63], *p* < 0.001). Therefore, there was no significant difference in the derived 1st DRR between the death and survival patients; however, there was a significantly higher 2nd DRR in the death patients than in the survival patients (1.92 [1.24–3.28] vs. 1.17 [0.83–1.61], *p* < 0.001). These results showed that a higher 2nd DRR was associated with higher mortality risk. This elevated DRR was mainly attributed to an elevated AST level in the second measurement of the patients who died.

Regarding the scores of various prognosis prediction models (Table 3), the dead patients presented significant differences in the scores of all prognosis prediction models compared to the survival patients (all *p* < 0.001).

### 3.2. Analysis of the Plotted ROC Curve

**Figure 2:**
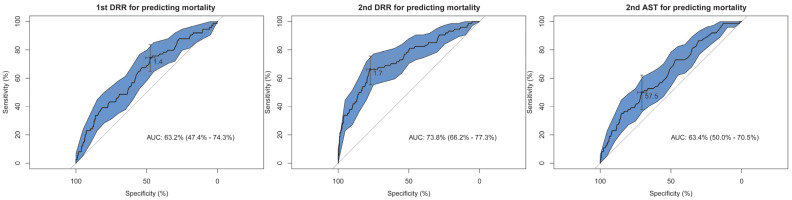
The receiver operating characteristic curves and the area under the curve (AUCROC) of the 1st and 2nd DRR to predict the mortality of the adult trauma patients in the intensive care unit.

**Figure 3:**
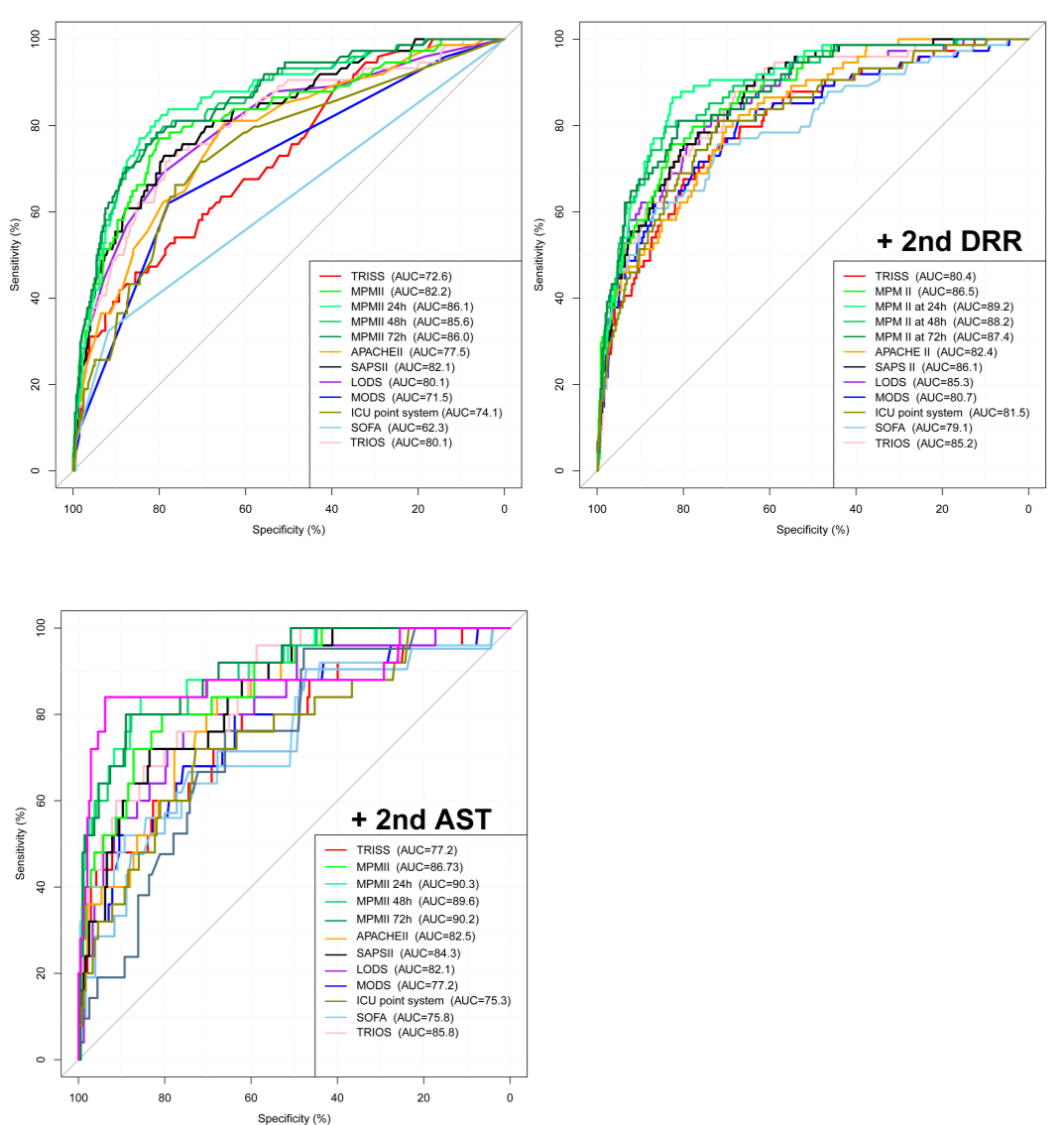
The receiver operating characteristic curves and the area under the curve (AUCROC) of the prognosis prediction models in predicting mortality (left above figure) and those curves with inclusion of the 2nd DRR (right above figure) or 2nd AST (left lower figure) as an additional variable in the mortality outcome prediction.

According to the ROC curve analysis (Figure 2), the 1st DRR of 1.4 and a 2nd DRR of 1.7 were identified as the cutoff points with the highest AUC of 63.2% (47.4–74.3%) and 73.8% (66.2–77.3%), respectively. In addition, the 2nd AST of 57.5 was identified as the cutoff point to predict the mortality (AUC 63.4%, 50.0–70.5%) (Figure 2). The inclusion of the 2nd DRR as an additional variable in the prognosis prediction models via logistic regression generated a significantly higher AUC for predicting mortality in all models (Figure 3), including TRISS (AUC, 72.6–80.4%), MPM II (82.2–86.5%), MPM_24_ II (86.1–89.2%), MPM_48_ II (85.6–88.2%), MPM_72_ II (86.0–87.4%), APACHE II (77.5–82.4%), SAPS II (82.1–86.1%), LODS (80.1–85.3%), MODS (71.5–80.7%), 24-h ICU point system (74.1–81.5%), SOFA (62.3–79.1%), and TRIOS (80.1–85.2%) (all *p* < 0.001). The inclusion of the 2nd AST as an additional variable in the prognosis prediction models generated a significantly higher AUC for predicting mortality in all models (Figure 3), but the AUCs of all prognosis prediction models were lower than those with inclusion of the 2nd DRR as an additional variable. The inclusion of 2nd DRR into MPM_24_ II had the highest AUC (89.2%), followed by MPM_48_ II (88.2%), and MPM_72_ II (87.4%).

## 4. Discussion

This study revealed that a higher 2nd DRR was associated with a higher mortality risk for trauma patients in the ICU, and the inclusion of the 2nd DRR as an additional variable in those prognosis prediction models generated significantly better prediction performance in all models. The inclusion of 2nd DRR into MPM24 II had the highest AUC (89.2%). The MPM II uses data on heath condition (medical or unscheduled surgical admission), pre-existing illness (such as metastatic neoplasm and cirrhosis), acute diagnosis (such as infection, intracranial mass effect, and coma), physiological variables (such as Cr levels, urine output, and partial pressure of oxygen), laboratory data (prothrombin time), and some other variables (such as mechanical ventilation and use of vasoactive drugs) [35,37]. Although MPM II had already considered the cirrhosis condition of the patients, the inclusion of 2nd DRR still can increase the AUC of prediction from 86.1% to 89.2%, indicating the relative change of AST and ALT help to assess the outcome of the patients with major trauma. In addition, the elevated DRR was mainly attributed to an elevated AST level in the second measurement of the death patients. Notably, although both AST and ALT are involved in aerobic glycolysis, catalyzing nucleotide and nonessential amino acids [46,47,48], an isolated elevation of AST values indicates a non-hepatic source of AST from the injury to non-liver cells, particularly those cells containing mitochondria [3]. Elevated AST levels, but not ALT, led to a higher DRR and indicated mitochondrial dysfunction upon oxidative stress [3,49,50]. Therefore, it has also been reported that in many cancers utilizing glucose, DRR is related to the metabolism of malignancies [51].

How high the DRR in a single measurement would indicate a worse outcome may vary greatly, depending on the illness studied. For example, a DRR ≥ 1.2 specify a higher mortality risk for patients with acute myocardial infarction [17]. A DRR ≥ 1.5 provide a significant postoperative prognostic factor for patients with renal cell carcinoma [52]. In patients with peripheral arterial occlusive disease, a DRR > 1.67 was associated with two-fold odds of risk for critical limb ischemia [53]. For patients with distal cholangiocarcinoma, a DRR > 2.0 was identified as a prognostic indicator [54]. In this study, a 2nd DRR of 1.7 was identified as the cutoff point to stratify the mortality risk of the patients.

Theoretically, the variables input into the mortality prediction model includes four classifications: age, acute diagnosis, pre-existing comorbidities, and physiological changes. The studied models of SAPS II, LODS, MODS, SOFA, and TRIOS took the bilirubin level into the algorithm for outcome prediction, while the MPM II model used cirrhosis as a weighted score for outcome prediction. TRISS, APACHE II, and 24-h ICU point system did not include any variable regarding liver function in the outcome prediction. However, in this study, the inclusion of the 2nd DRR as an additional variable in the prognosis prediction models generated significantly better prediction performance in all models, implying that liver function did matter in influencing the mortality outcome and the 2nd DRR, which was measured days after admission into the ICU, may be more sensitive than the bilirubin level in the mortality prediction, particularly considering that there was no significant difference in the bilirubin level of the death and survival patients at admission into the ICU.

This study had some limitations. First, this retrospective study may have led to selection bias in the outcome assessment. Second, some selection bias may exist in the study because the primary outcome measured in-hospital mortality but did not include death declared on arrival at the emergency room and death in long-term mortality. Moreover, those who died within 3 days after admission to the ICU did not have a second measurement of liver enzymes. Third, the extent of muscle injury and the interventions or management, such as surgery, massive blood transfusion and resuscitation, may have led to a bias in the measurement of liver function; however, the influence was unknown. Fourth, in the presence of undetected liver diseases or drug use, the serum levels of AST and ALT, as well as the derived DRR, may be disturbed, leading to bias in the outcome assessment. Various pre-existed comorbidity conditions may also result in the bias in the outcome assessment. Finally, the study population was limited to a single urban trauma center without confirmation in other regions.

## 5. Conclusions

This study revealed that the inclusion of the additional variable of DRR, which was measured from day 3 to 7 after ICU admission, significantly increased the prediction performance in all the studied prognosis prediction models.

## Figures and Tables

**Table 1:** Categorical variables of patient and injury characteristics of the death and survival adult trauma patients who were admitted into the intensive care unit.

Variables		Total	Mortality		*p*-Value
		(*n* = 888)	No (*n* = 814)	Yes (*n* = 74)	
Sex	Female	289 (32.6%)	265 (32.6%)	24 (32.4%)	>0.999
	Male	599 (67.5%)	549 (67.4%)	50 (67.6%)	
HTN	No	597 (67.2%)	558 (68.6%)	39 (52.7%)	0.007
	Yes	291 (32.8%)	256 (31.5%)	35 (47.3%)	
CAD	No	817 (92.0%)	754 (92.6%)	63 (85.1%)	0.040
	Yes	71 (8.0%)	60 (7.4%)	11 (14.9%)	
ESRD	No	868 (97.8%)	802 (98.5%)	66 (89.2%)	<0.001
	Yes	20 (2.3%)	12 (1.5%)	8 (10.8%)	
CVA	No	854 (96.2%)	785 (96.4%)	69 (93.2%)	0.194
	Yes	34 (3.8%)	29 (3.6%)	5 (6.8%)	
DM	No	718 (80.9%)	663 (81.5%)	55 (74.3%)	0.163
	Yes	170 (19.1%)	151 (18.6%)	19 (25.7%)	
AIS (Head)	0	225 (25.3%)	214 (26.3%)	11 (14.9%)	<0.001
	1	8 (0.9%)	8 (1.0%)	0 (0.0%)	
	2	18 (2.0%)	17 (2.1%)	1 (1.4%)	
	3	76 (8.6%)	72 (8.9%)	4 (5.4%)	
	4	400 (45.1%)	376 (46.2%)	24 (32.4%)	
	5	160 (18.0%)	127 (15.6%)	33 (44.6%)	
	6	1 (0.1%)	0 (0.0%)	1 (1.4%)	
AIS (Face)	0	715 (80.5%)	651 (80.0%)	64 (86.5%)	0.357
	1	20 (2.3%)	20 (2.5%)	0 (0.0%)	
	2	146 (16.4%)	136 (16.7%)	10 (13.5%)	
	3	7 (0.8%)	7 (0.9%)	0 (0.0%)	
AIS (Thorax)	0	569 (64.1%)	524 (64.4%)	45 (60.8%)	0.523
	1	25 (2.8%)	25 (3.1%)	0 (0.0%)	
	2	56 (6.3%)	51 (6.3%)	5 (6.8%)	
	3	134 (15.1%)	122 (15.0%)	12 (16.2%)	
	4	91 (10.3%)	81 (10.0%)	10 (13.5%)	
	5	13 (1.5%)	11 (1.4%)	2 (2.7%)	
AIS (Abdomen)	0	668 (75.2%)	605 (74.3%)	63 (85.1%)	0.040
	2	77 (8.7%)	75 (9.2%)	2 (2.7%)	
	3	80 (9.0%)	78 (9.6%)	2 (2.7%)	
	4	43 (4.8%)	37 (4.6%)	6 (8.1%)	
	5	20 (2.3%)	19 (2.3%)	1 (1.4%)	
AIS (Extremity)	0	523 (58.9%)	472 (58.0%)	51 (68.9%)	0.528
	1	5 (0.6%)	5 (0.6%)	0 (0.0%)	
	2	214 (24.1%)	202 (24.8%)	12 (16.2%)	
	3	129 (14.5%)	119 (14.6%)	10 (13.5%)	
	4	16 (1.8%)	15 (1.8%)	1 (1.4%)	
	5	1 (0.1%)	1 (0.1%)	0 (0.0%)	
AIS (External)	0	848 (95.5%)	779 (95.7%)	69 (93.2%)	0.667
	1	28 (3.2%)	25 (3.1%)	3 (4.1%)	
	2	7 (0.8%)	6 (0.7%)	1 (1.4%)	
	3	1 (0.1%)	1 (0.1%)	0 (0.0%)	
	4	1 (0.1%)	1 (0.1%)	0 (0.0%)	
	5	3 (0.3%)	2 (0.3%)	1 (1.4%)	

AIS = abbreviated injury scale; CAD = coronary artery disease; CVA = cerebral vascular accident; DM = diabetes mellitus; ESRD = end-stage renal disease; HTN = hypertension.

**Table 2:** Continuous variables of patient and injury characteristics of the death and survival adult trauma patients who were admitted into the intensive care unit.

Variables	Total	Mortality		*p*-Value
	(*n* = 888)	No (*n* = 814)	Yes (*n* = 74)	
Age (years)	59 [40, 70]	58 [39, 69]	71 [58, 78]	<0.001
BMI	23.9 [21.4, 27.1]	23.9 [21.4, 27.1]	23.8 [21.4, 28.0]	0.861
Temperature (°C)	36.5 [36.1, 37.0]	36.5 [36.1, 37.0]	36.2 [36.0, 36.7]	0.001
HR (beats/min)	92 [80, 106]	92 [80, 106]	98 [86, 112]	0.030
SBP (mmHg)	140 [122, 156]	139 [123, 156]	141 [116, 158]	0.675
RR (times/min)	19 [18, 20]	19 [18, 20]	20 [17, 22]	0.932
GCS	11 [7, 15]	11 [8, 15]	6 [3, 11]	<0.001
ISS	20 [16, 25]	20 [16, 25]	25 [19, 29]	<0.001
Glucose (mg/dL)	159 [133, 205]	158 [132, 198]	197 [157, 250]	<0.001
HCO_3_ (meq/L)	21.7 [19.3, 23.6]	21.8 [19.4, 23.7]	20.4 [18.5, 22.5]	0.003
Na (mEq/L)	138 [136, 140]	138 [136, 140]	139 [136, 141]	0.599
K (mEq/L)	3.6 [3.2, 3.9]	3.6 [3.3, 3.9]	3.7 [3.1, 4.2]	0.742
RBC (10^6^/μL)	4.4 [3.9, 4.9]	4.4 [3.9, 4.9]	4.4 [3.9, 4.8]	0.864
WBC (10^3^/μL)	11.6 [8.4, 15.7]	11.6 [8.6, 15.5]	11.4 [7.5, 16.0]	0.572
Neutrophil (%)	79.5 [68.1, 85.5]	79.3 [68.0, 85.5]	81.1 [69.5, 85.2]	0.702
Hb (g/dL)	13.1 [11.5, 14.4]	13.1 [11.6, 14.4]	13.0 [10.9, 14.6]	0.665
Hct (%)	39.2 [34.8, 42.9]	39.2 [35.1, 42.9]	39.7 [33.7, 42.6]	0.594
Platelets (10^3^/μL)	215 [169, 266]	215 [169, 266]	215 [172, 265]	0.729
INR	1.05 [1.01, 1.12]	1.05 [1.00, 1.12]	1.09 [1.03, 1.20]	<0.001
BUN (mg/dL)	15 [11, 19]	14 [11, 19]	19 [13, 26]	<0.001
Cr (mg/dL)	0.96 [0.76, 1.21]	0.94 [0.75, 1.19]	1.19 [0.95, 2.06]	<0.001
BUN/Cr	14.97 [11.39, 19.23]	15.04 [11.46, 19.25]	13.69 [10.72, 19.09]	0.192
Albumin (g/dL)	3.3 [2.9, 3.7]	3.4 [2.9, 3.8]	2.9 [2.5, 3.6]	<0.001
Bilirubin (mg/dL)	0.8 [0.6, 1.1]	0.8 [0.6, 1.1]	0.8 [0.5, 1.4]	0.826
1st AST (U/L)	53 [33, 128]	53 [33, 128]	54 [34, 118]	0.768
1st ALT (U/L)	37 [22, 88]	37 [22, 92]	29 [19, 61]	0.050
2nd AST (U/L)	40 [26, 66]	39 [26, 63]	57 [32, 107]	<0.001
2nd ALT (U/L)	34 [20, 62]	34 [20, 62]	25 [16, 56]	0.066
1st DRR	1.49 [1.19, 1.88]	1.46 [1.18, 1.85]	1.66 [1.41, 2.26]	<0.001
2nd DRR	1.22 [0.85, 1.69]	1.17 [0.83, 1.61]	1.92 [1.24, 3.28]	<0.001

ALT = alanine aminotransferase; AST = Aspartate transaminase; BMI = body mass index; BUN = blood urea nitrogen; Cr = creatinine; DRR = De Ritis ratio; HCO_3_ = bicarbonate; HR = heart rate; GCS = Glasgow coma scale; Hb = hemoglobin; Hct = hematocrit; INR = international normalized ratio; K = potassium; Na = sodium; ISS = injury severity score; RBC = red blood cells; RR = respiratory rate; SBP = systolic blood pressure; WBC = white blood cells. These continuous data was expressed with median and interquartile range.

**Table 3:** The scores of various prognosis prediction algorithms for the death and survival adult trauma patients who were admitted into the intensive care unit.

Variables	Total	Mortality		*p*-Value
	(*n* = 888)	No (*n* = 814)	Yes (*n* = 74)	
TRISS	0.93 [0.79, 0.97]	0.93 [0.82, 0.97]	0.79 [0.27, 0.93]	<0.001
MPM II	11.9 [7.1, 22.5]	11.4 [6.9, 19.2]	44.4 [23.5, 69.9]	<0.001
MPM_24_ II	7.8 [4.4, 14.1]	7.2 [4.1, 12.5]	29.4 [16.7, 49.9]	<0.001
MPM_48_ II	10.2 [5.6, 17.8]	9.4 [5.2, 15.7]	34.9 [19.5, 56.4]	<0.001
MPM_72_ II	12.1 [6.6, 21.3]	10.8 [6.1, 18.2]	40.6 [22.7, 60.2]	<0.001
APACHE II	13 [8, 18]	12 [8, 18]	21 [16, 26]	<0.001
SAPS II	28 [19, 38]	27 [18, 37]	49 [35, 56]	<0.001
LODS	2 [1, 4]	2 [1, 4]	6 [4, 8]	<0.001
MODS	3 [1, 5]	2 [1, 4]	5 [4, 6]	<0.001
24 h ICU point system	1 [0, 4]	1 [0, 3]	4 [2, 6]	<0.001
SOFA	3 [1, 5]	3 [1, 5]	5 [4, 8]	<0.001
TRIOS	8.7 [4.3, 17.7]	7.6 [4.2, 15.0]	23.6 [14.8, 41.5]	<0.001

APACHE = the Acute Physiology and Chronic Health Evaluation; ICU = intensive care unit; LODS = Logistic Organ Dysfunction System; MODS = Multiple Organs Dysfunction Score; MPM = Mortality Prediction Model; SAPS = Simplified Acute Physiology Score; SOFA = Sequential Organ Failure Assessment; TRIOS = Three-Day Recalibrating ICU Outcomes; TRISS = The Trauma Score and Injury Severity Score.

## Data Availability

Not applicable.
